# Is decision-making impairment an endophenotype of anorexia nervosa?

**DOI:** 10.1192/j.eurpsy.2022.2327

**Published:** 2022-10-21

**Authors:** Laura Di Lodovico, Audrey Versini, Mathieu Lachatre, Jacopo Marcheselli, Nicolas Ramoz, Philip Gorwood

**Affiliations:** 1 Clinique des Maladies Mentales et de l’Encéphale, Hopital Sainte-Anne, GHU Paris Psychiatrie et Neurosciences, Paris, France; 2 Université Paris Cité, Institute of Psychiatry and Neuroscience of Paris (IPNP), INSERM U1266, Paris, France; 3 ARIA (Now SUEZ), Boulogne-Billancourt, France; 4 International School for Advanced Studies (SISSA), Trieste, Italy

**Keywords:** Anorexia nervosa, Bayesian, decision-making, endophenotype, Iowa gambling task

## Abstract

**Background:**

Patients with anorexia nervosa (AN) show impaired decision-making ability, but it is still unclear if this is a trait marker (i.e., being associated with AN at any stage of the disease) or a state parameter of the disease (i.e., being present only in acutely ill patients), and if it has endophenotypic characteristics. The aim of this study was to determine the endophenotypic, and state- or trait-associated nature of decision-making impairment in AN.

**Methods:**

Ninety-one patients with acute AN (A-AN), 90 unaffected relatives (UR), 23 patients remitted from AN (R-AN), and 204 healthy controls (HC) carried out the Iowa gambling task (IGT). Prospective valence learning (PVL) model was employed to distinguish the cognitive dimensions underlying the decision-making process, that is, learning, consistency, feedback sensitivity, and loss aversion. IGT performance and decision-making dimensions were compared among groups to assess whether they had endophenotypic (i.e., being present in A-AN, UR, and R-AN, but not in HC) and/or trait-associated features (i.e., present in A-AN and R-AN but not in HC).

**Results:**

Patients with A-AN had lower performance at the IGT (*p* < 0.01), while UR, R-AN, and HC had comparable results. PVL-feedback sensitivity was lower in patients with R-AN and A-AN than in HC (*p* < 0.01).

**Conclusions:**

Alteration of decision-making ability did not show endophenotypic features. Impaired decision-making seems a state-associated characteristic of AN, resulting from the interplay between trait-associated low feedback sensitivity and state-associated features of the disease.

## Introduction

Anorexia nervosa (AN) is a severe psychiatric illness characterized by an intense fear of gaining weight and a distortion of body image, leading to malnutrition that is often denied. Weight controlling strategies such as food restriction, purging (vomiting and laxative misuse), and inappropriate physical exercise are its core behavioral symptoms [1–3]. The paradoxical persistence of these behaviors along with ongoing malnutrition, and in spite of self-damage [4], mirrors a tendency to prefer the immediate effect of these maladaptive behaviors over their long-term negative consequences [5, 6].

Tasks exploring decision-making ability have shown impaired performances in patients with AN [7]. This inability to make the most advantageous decisions could be underpinned by different factors, such as a dysregulation of reward processing [7–9], anxiety, poor enteroception, intolerance to uncertainty [10–12], and cognitive rigidity [13]. If decision-making impairment is widely recognized as a cognitive feature of AN, it is still debated whether it represents an endophenotype (i.e., associated with the illness, heritable, trait-associated, and present in unaffected family members of patients at higher rates than in the general population) [14], and whether it is a state-associated alteration (i.e., being explained by acute symptoms and malnutrition) or trait-associated feature (associated to illness and present at any stage of the disease, independently from acute symptoms). Past explorations of decision-making ability in AN provide contrasting findings and make difficult to close the debate. Two studies on remitted patients found normal decision-making in patients after weight restoration [15, 16], suggesting this alteration as state-associated, while others support a trait-associated nature for decision-making alteration, as independent from nutritional status [7, 17, 18]. The only study including healthy relatives proposed impaired decision-making as an endophenotype of AN [19].

Most of the previous studies limited the exploration to the raw scores of the neuropsychological test used, that is, in most cases, the Iowa gambling task (IGT) [20]. Raw IGT scores measure risk-related decision-making under conditions of uncertainty [21], but do not identify the psychological processes underneath [22]. Cognitive process modeling, informed by cognitive neuroscience and Bayesian theories [23], allows a more precise analysis of the multiple processes involved in a cognitive task by deconstructing task performance into theorized underlying psychological processes [24].

The prospect valence learning (PVL) model is a performant and widely used model to explain the mechanisms underlying decision-making [25], already employed in studies on patients with AN [24, 26].

Previous studies employing the PVL model found discrepant results [24, 26, 27]. Small samples of patients with AN [24, 27] or at risk of AN [26] were compared with patients with bulimia nervosa [24], and healthy controls (HC) [24, 26, 27]. Patients with AN reported an alteration of the decision-making parameters identified by the PVL, such as lower learning scores [24, 26], response consistency [26], and loss aversion [27]. Neither patients remitted from AN, nor unaffected relatives (UR) of patients have ever been tested with this model.

Several questions on decision-making in AN are thus still open. It is still to be determined whether altered decision-making is an endophenotype of AN, and whether it is associated with the illness as a trait or a state. Moreover, nothing is known about the endophenotypic, trait, or state nature of the cognitive dimensions underlying decision-making in AN.

In this context, studies addressing the nature of decision-making alteration in AN are still necessary, and should overcome the limitations of small sample sizes, and analyses limited to raw IGT scores. Another problem is represented by the heterogeneity of the cognitive models employed. Comparability with previous studies will be ensured by choosing one of the already-used cognitive models in previous research.

The aim of the current study was to assess whether impaired decision-making ability is an endophenotype of AN, and whether it is a state- or trait-associated feature of the illness. We also assumed different decision-making profiles in patients with acute AN (A-AN), distinguishing them from patients with remitted AN, UR, and HC.

## Methods

### Participants

The present study utilizes data collected as part of a monocentric, cross-sectional open trial. Four hundred and eleven participants were offered the possibility to participate in this protocol. Three were excluded, two because of previous knowledge of the test and one because of registration failure of results. In total, 408 participants were included in this study, of whom 91 were patients with A-AN, 90 were UR of patients with AN (UR) unrelated to the patients included (79 mothers, 11 sisters), 204 were HC subjects and 23 were patients remitted from AN (R-AN).

Patients with A-AN and R-AN were recruited from the inpatient and outpatient unit of the *Clinique des Maladies Mentales et de l’Encéphale* of Sainte Anne hospital in Paris. Inclusion criteria for patients with A-AN were a diagnosis of A-AN according to the DSM-5 [1] and the Diagnostic interview for genetic studies (DIGS) [28, 29], and body mass index (BMI) < 18.5 kg/m^2^. Patients with R-AN were included if they had a diagnosis of past AN at the DIGS and of remitted AN at the DSM-5, with a BMI > 18.5 kg/m^2^. All patients were assessed only once.

UR were recruited from family groups and family interviews provided in the same hospital. HC were recruited at universities, at research laboratories and by ear-to-mouth and should report no family history of eating disorders. Exclusion criteria for both groups were BMI < 18.5 kg/m^2^ and a diagnosis of past or present personal history of eating disorders according to the DIGS.

All participants were Caucasian females. Previous knowledge of the IGT, impaired performance at the National Adult Reading Test (NART), poor understanding of French language, and a diagnosis of psychosis and/or bipolar disorder represented exclusion criteria for all groups.

All study procedures were approved by the *Ile-de-France III* ethics committee (#A01636-49) and the *Commission Nationale de l’Informatique et des Libertés.* In accordance with the Helsinki declaration, written informed consent was obtained from each participant before inclusion.

### Variables

Age, BMI, and NART score [30] were collected for each participant. All participants were measured in height and weight after night starvation, and BMI was calculated in kg/m^2^.

The DIGS is a semistructured interview developed to collect comprehensive databases of psychiatric symptoms, signs, current, and lifetime psychiatric history. This instrument showed good validity, interrater, and test–retest reliability in all translated versions. Moreover, it proved capable features of collecting both cross-sectional and longitudinal data with clear diagnostic criteria, and extensive information on the course and chronology of comorbid conditions, thereby reducing their contamination on the psychiatric phenotypes of interest [28, 29]. Illness duration, lifetime BMI, and duration of remission from AN were collected by the DIGS, given their potential impact on cognitive performance [31].

The NART consists of a list of irregularly spelled words, graded in difficulty [32]. This test is adopted as a screening measure for verbal intelligence, as the number of words pronounced correctly shows a high correlation with the Wechsler Adult Intelligence Scale [33] verbal and total IQ scores (*r* = 0.80 and *r* = 0.77, respectively).

Eating disorder psychopathology was assessed by the Eating disorder inventory-2 (EDI-2), using a validated French version [34]. This widely used instrument explores attitudinal and behavioral dimensions relevant to eating disorders. Internal consistency is >0.60 in healthy subjects and >0.80 in patients with AN for the eight EDI original dimensions [35], and 0.65 to 0.75 for the three additional dimensions [36].

Since previous studies of decision-making on eating-disordered samples found a correlation of poor decision-making with anxiety [37] and impulsivity [38], we employed two scales to measure these dimensions.

Anxiety was measured by the French version of the State–Trait Anxiety Inventory-Y (STAI-Y) [39]. The STAI-Y is a self-reported inventory composed of 40 questions based on a 4-point Likert scale. The STAI-Y measures two types of anxiety—state anxiety, or situational anxiety, and trait anxiety, as a personal characteristic. Higher scores are positively correlated with higher levels of anxiety [40]. This scale shows good internal consistency both for the state (Cronbach’s alpha 0.90) and the trait (0.91) aspects [39].

Impulsivity was assessed by the French version of the Barratt Impulsivity Scale (BIS-10) [41]. The BIS-10 is a gold standard measure of impulsiveness by the analysis of the subtracts of cognitive impulsiveness, motor impulsiveness, and nonplanning impulsiveness [42]. Internal consistency for the total score of the French-validated version is 0.82 [41].

### Iowa gambling task

The most widely used instrument to assess decision-making is the IGT. This test profiles the capacity to learn from past experience, the tendency for risk-taking, and impulsive behavior, characterized by the preference of immediate gains despite negative consequences. The IGT assesses decision-making alterations [20], simulating real-life decision-making by factoring reward and punishment [43]. Players have to choose between cards providing immediate rewards, with the risk of important losses, and cards associated with moderate gains, but also moderate losses.

A computerized version of the IGT [44] was used to evaluate decision-making. Participants have to choose from 4 decks of 25 cards (100 cards in total) presented on a computer monitor, with the aim of achieving monetary gain. While decks A and B give large immediate gains but also high losses, (high-risk), decks C and D give smaller rewards but also lower losses (low-risk). No information is provided on the content of each deck.

The 100 experimental trials can be divided into blocks of 20 trials each, where the block net score is calculated by subtracting the number of trials choosing advantageous decks, minus the number of trials choosing disadvantageous decks [(*C* + *D*) − (*A* + *B*)]. Performance is also assessed by the net score calculated on the 100 sorts (net IGT score).

### Cognitive modeling analysis by PVL model

This model was developed from the expectancy valence model (EVL), that assumes “attention to winning,” “recency,” and “consistency” as the three determinants of IGT performance [45]. The PVL has the advantage to account for the influence of the frequency of gains and losses on the formation of expectancy, and to be more performant in short-term prediction and more fit for complex choice behaviors [21, 25]. In this model, the motivational process is analyzed by two proxies: “aversion to losses” and “sensitivity to feedback” [46]. Deck selection in each trial of the IGT is based on the expectancy of valence, based on learning on the experience of the previous gains and losses [24]. The expectancy of valence [*u*(*t*)] is formed by the ensemble of the magnitude of gains/losses, aversion to loss, and sensitivity to feedback as described by the equation, where *x*(*t*) is the net gain on the *t*th trial.

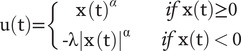



*x*(*t*)*^α^* determines the feedback sensitivity (*α*) (ranging from 0 to 1): the nonlinear relationship between the magnitude of the valence expected and that of the actual gain or loss. The closer to 1, the more proportional the expectance to the actual gain/loss. Loss aversion (*λ*) (0 to 5) measures the tendency for losses to influence the expectancy valence as opposed to gains. Values higher than 1 indicate greater sensitivity to losses than to gains. In addition to the *α* and *λ* parameters present in the equation, learning (*A*) (0 to 1) indicates at what degree the earlier experience influences the current expectancy valence. Response consistency (*c*) (0 to 5) represents the influence of expectancy on deck choice as opposite to random deck selection.

The four parameters of the PVL model were estimated by using the computational model developed and described by Anh et al. [47], who provided an R package hBayesDM (hierarchical Bayesian modeling of Decision-Making tasks) [48, 49]. This package offers a hierarchical Bayesian modeling which implements a Markov Chain Monte Carlo (MCMC) algorithm called Hamiltonian Monte Carlo (HMC). A more comprehensive description of HMC can be found at (https://mc-stan.org/documentation/) and [50]. Using the HMC sampling scheme, 1000 samples were drawn after a 800-sample burn-in with four chains.

### Statistical analysis

Normality of distribution was assessed by the inspection of skewedness and kurtosis. Values of skewedness between −2 and + 2, and values of kurtosis between −7 and + 7, were considered acceptable to prove normal univariate distribution [51, 52].

A-AN, R-AN, UR, and HC groups were compared on sociodemographic and clinical variables by analyses of variance (ANOVAs) with Bonferroni corrections for multiple testing.

The following decision-making indicators were compared between the four groups: IGT scores, PVL-A (learning), PVL-alpha (feedback sensitivity), PVL-C (consistency), and PVL-lambda (loss aversion), and included in the analysis of covariance (ANCOVA) as dependent variables. Age, intellectual level (NART score), impulsivity (BIS-10 score), and state anxiety (STAI-A score) were included as covariates in the model [12, 18, 37, 38, 53].

All tests were submitted to post hoc Bonferroni correction for multiple testing. A variable distinguishing patients and UR from HC would be retained as an endophenotype. One distinguishing A-AN and R-AN from UR and HC would be considered as trait-associated.

To rule out the potential role of depression [54], all analyses were repeated after excluding all participants with a diagnosis of depression at the DIGS. The effect of psychotropic drugs on cognitive performance was assessed by comparing IGT performance of patients with versus without psychotropic medications.

All statistical analyses were performed by SPSS (IBM Corp. Released 1989, 2021. IBM SPSS Statistics for Macintosh, Version 28.0.0.0 (190) Armonk, NY: IBM Corp).

## Results

### Descriptive results

All data were normally distributed. Table 1 presents sample characteristics, IGT performance, and PVL model parameters for each group.

**Table 1.** Clinical, demographic, psychometric characteristics, and IGT parameters of patients with acute anorexia nervosa (A-AN), healthy controls (HC) unaffected relatives of patients with AN (UR) and patients remitted from AN (R-AN).

	A-AN (*n* = 91)		HC (*n* = 204)		UR (*n* = 90)		R-AN (*n* = 23)		Four-group comparison		Post hoc comparisons
	Mean	SD	Mean	SD	Mean	SD	Mean	SD	*F*(3,407)	*p* value	
Age	27.54	7.34	37.60	15.68	52.12	11.49	29.52	4.54	58.91	<0.01	UR > HC > R-AN>A-AN
BMI	16.07	1.63	22.39	3.30	22.82	3.91	20.17	1.23	104.68	<0.01	A-AN<R-AN<HC, A-AN<R-AN<UR
Maximum lifetime BMI	21.75	3.51	23.85	3.60	24.31	4.39	23.70	3.54	12.52	<0.01	A-AN<HC, A-AN<UR, A-AN<R-AN
Minimum lifetime BMI	13.07	1.92	19.06	2.23	18.07	2.00	13.74	1.48	160.89	<0.01	A-AN<HC, A-AN<UR, R-AN<HC, R-AN<UR
EDI-2 score	202.00	94.39	46.62	25.57	87.00	29.98	90.00	35.20	155.63	<0.01	A-AN>R-AN>HC, A-AN>R-AN>UR
NART score	25.94	4.22	26.41	5.11	26.77	5.64	29.04	3.77	2.30	0.06	
STAI-A (state)	41.05	11.14	28.15	6.18	30.02	8.01	33.17	9.62	53.85	<0.01	A-AN>R-AN>HC, A-AN>UR
STAI-B (trait)	58.00	10.40	37.24	8.42	40.99	10.28	42.96	11.62	100.71	<0.01	A-AN>R-AN>UR > HC
BIS-10	47.56	15.31	49.76	13.52	48.04	12.33	49.00	13.28	0.47	0.71	
IGT net score 1–20*	−3.68	0.77	−2.27	0.49	−2.87	0.49	−5.04	1.45	2.40	0.07	
IGT net score 21–40*	1.95	0.89	3.41	0.56	4.38	0.94	6.61	1.67	2.58	0.05	
IGT net score 41–60*	2.69	1.00	5.71	0.63	5.95	1.06	6.62	1.88	2.27	0.08	
IGT net score 61–80*	1.62	1.15	5.46	0.72	7.18	1.22	5.86	2.16	3.39	0.02	A-AN<HC, A-AN<UR
IGT net score 81–100*	2.35	1.18	5.66	0.74	6.43	1.24	6.77	2.21	0.79	0.50	
IGT total net score*	10.88	27.61	18.19	30.32	13.79	30.33	25.65	32.23	3.63	0.01	A-AN<HC, A-AN<UR
PVL-A (learning)*	0.53	0.25	0.50	0.22	0.48	0.25	0.57	0.17	0.54	0.66	
PVL-Alpha (feedback sensitivity)*	0.16	0.03	0.20	0.12	0.30	0.09	0.09	0.01	32.49	<0.01	UR > HC > A-AN>R-AN
PVL-C (consistency)*	0.67	0.30	0.74	0.31	0.76	0.34	0.78	0.34	0.82	0.49	
PVL-Lambda (loss aversion)*	1.57	1.69	1.16	1.73	0.94	1.04	0.41	0.25	3.34	0.02	A-AN>R-AN

*Note:* The variables marked with an asterisk were calculated by ANCOVAs with age, NART, BIS-10, and STAI-A scores as covariates.

*Abbreviations:* BIS, Barratt impulsivity scale; BMI, body mass index; EDI, eating disorder inventory; IGT, Iowa gambling test; NART, national adult reading test; PVL, prospect valence learning; STAI, state–trait anxiety inventory.

Average illness duration was of 87.81 ± 78.64 months for A-AN patients, and of 63.91 ± 48.36 months for the R-AN group, with remission duration of 77.21 ± 83.36 months. Fifty patients had daily benzodiazepine administration, 39 had antidepressants, and 17 had antipsychotics (Supplementary Table S1).

Age was significantly different across groups, and patients had the highest levels of anxiety (*p* < 0.01). No difference was found in impulsivity among groups.

### IGT performance and PVL model parameters

**Figure 1.**
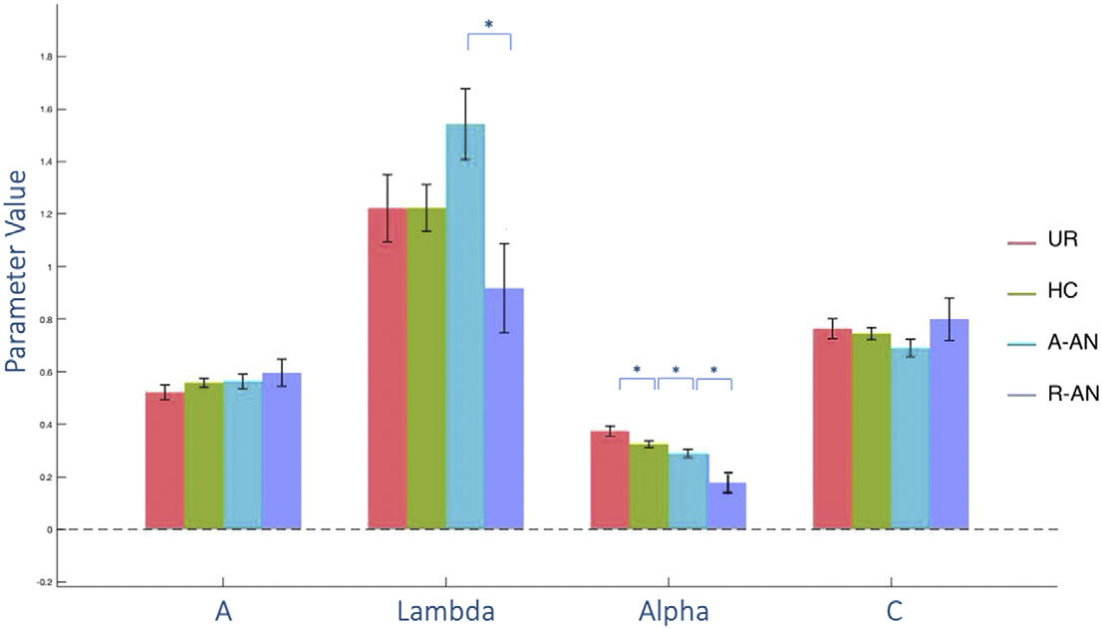
Decision-making parameters according to the prospect valence learning model across four groups of patients with acute anorexia nervosa (A-AN), remitted anorexia nervosa (R-AN), unaffected relatives (UR), and healthy controls (HC). The four decision-making parameters are: learning (*A*), feedback sensitivity (alpha), consistency (*C*), and loss aversion (lambda). A-AN is characterized by low feedback sensitivity. R-AN are characterized by low feedback sensitivity and lower loss aversion than A-AN. Significant inter-group differences are shown by asterisks (p < 0.05).

A-AN patients had the lowest total net IGT scores (*p* < 0.01). Feedback sensitivity (PVL-alpha) was significantly lower in A-AN than in HC, whereas no difference emerged for the three other PVL parameters (*p* > 0.05). A-AN patients also showed higher aversion to losses (PVL-lambda) than R-AN (*p* < 0.01) (Figure 1).

UR had comparable performance with HC on all parameters (*p* > 0.05), except for feedback sensitivity that was higher than in the three other groups (*p* < 0.01).

Like A-AN, R-AN had low values of feedback sensitivity (*p* < 0.01), but their performance at the IGT was comparable to HC (*p* > 0.05).

Significance of results was preserved after excluding the 22 patients with depression for the five-block IGT score (*F*(3,376) = 3.06, *p* = 0.03), the IGT total net score (*F*(3,376) = 2.97, *p* = 0.03), and the PVL parameter analyses (*F*(3,376) = 28.00, *p* < 0.01 for PVL-*alpha,* and *F*(3,376) =3.37, *p* = 0.02 for the PVL-*lambda*).

Benzodiazepines, antidepressants, and antipsychotics did not affect IGT performance in patients with AN (*p* > 0.05) (Supplementary Table S1). Supplementary Figure S1 shows mean IGT block scores for each group.

### Post hoc correlations

To explore whether any clinical indicator was related to IGT performance, we calculated post hoc correlations between total net IGT score and duration of illness (in A-AN and R-AN) and of remission (in R-AN), and minimum and current BMI (in the whole sample), controlling for age, NART, STAI-A, and BIS-10. We found no correlation between IGT performance and duration of illness (*r* = −0.08, *n* = 114, *p* = 0.42), of remission (*r* = 0.07, *n* = 23, *p* = 0.08), and minimum lifetime BMI (*r* = 0.07, *n* = 408, *p* = 0.79), and a significant correlation between total net IGT score and current BMI (*r* = 0.11, *n* = 408, *p* = 0.04).

## Discussion

This study explored whether decision-making deficits could represent a third cognitive endophenotype of AN and/or a trait-associated feature, by a cognitive modeling approach. The present results suggest decision-making impairment as a state-associated feature of AN, partially underpinned by a trait-associated diminution of feedback sensitivity.

This study dovetails with previous research confirming impaired performance at the IGT in patients with A-AN [7]. Normal performance of UR suggests that this impairment is not an endophenotype, respecting no criteria of an endophenotype as listed by Gottesman and Gould [14]. Moreover, in line with previous studies including remitted patients [15, 16], we found comparable IGT scores between R-AN and HC, suggesting decision-making impairment as a state-related feature of AN, unaffected in patients who previously suffered from AN and are at normal nutritional status.

The intriguing finding of low PVL-feedback sensitivity in remitted patients suggests this cognitive feature as trait-dependent in AN. Low sensitivity to feedback may underpin some alterations observed in patients with AN, such as the difficulty in discriminating between reward and punishment [9], and reduced feedback learning [55]. Altered responses to reward, and difficulties in discriminating positive from negative feedback, were already described in remitted patients [56], even though monetary reward processing was investigated by means of other cognitive tasks, such as the delay discounting test. Studies of functional magnetic resonance supporting this hypothesis showed a lack of differential activation in reward-related neural circuits in response to monetary wins and losses [57].

The coexistence of trait-associated cognitive dimensions underpinning decision-making, and of successful decision-making after remission from AN, may suggest the existence of compensatory mechanisms occurring at a normal nutritional status and/or relief from symptoms.

For instance, a functional magnetic resonance study on women recovered from AN found exaggerated activation in the caudate–dorsal striatum and in the “cognitive” cortical regions that project to this area, such as the dorsolateral prefrontal and the parietal cortex [57]. One could assume that the hyperactivation of these brain regions could represent a compensatory mechanism allowing increased cognitive control on decision-making in remitted patients. Additionally, remitted patients showed enhanced cognitive control allowing for “strategic” (as opposed to hedonic) approaches to improve the ratio of wins to losses, despite altered reward system activation to monetary stimuli [56, 58]. Learning capacity may also play a role in the improvement of decision-making and response to therapy [59]. Impaired IGT performance in A-AN could be interpreted as a consequence of weaker prefrontal efficiency secondary to malnutrition, and/or state-dependent neurobiological alterations such as reduced amygdala gray matter volume [60] and altered fronto-amygdalar response [61].

Post hoc analyses allowed an investigation of the clinical factors involved in decision-making performance, highlighting the role of poor nutritional status. The association between cognitive parameters of IGT performance and state markers of AN, already found in previous literature [27], suggests that the achievement of a normal nutritional state and long-term remission may reverse these deficits [15]. Further cognitive parameters that seem significantly different in acute versus remitted AN, such as high loss aversion, may play an additional role in decision-making alterations in A-AN by a differential evaluation of gains and losses.

This study presents discrepant results from those that previously applied the PVL model in AN [24, 26]. For example, higher sensitivity to feedback was found in patients with BN but not with AN, who were characterized by reduced learning ability [24]. Verharen et al. [27], had found low loss aversion in patients with AN. Some explanations could be the use of a different cognitive model, or a different approach to the task: as underlined by the authors, the values of the cognitive parameter of consistency rather suggested a more random choice of decks as the session progressed [27], reflecting fatigue or boredom.

This work proposes a neurocognitive interpretation of the persistence of symptoms of AN, as a result of the progressive deterioration of decision-making along with illness progression. Disadvantageous behaviors, such as pursuing weight-loss behaviors despite their long-term negative effects on health, could be seen as a result of this impairment of decision-making and reward evaluation [62]. For instance, impaired feedback sensitivity could explain the pursuit of certain behavioral excesses, devoted to weight loss, and insensitive to some consequences that healthy subjects would consider punishing, such as hunger and fatigue [63].

This research presents a set of limitations. First, this protocol did not include any quantitative assessment of depression besides eliminating qualitative major depressive episodes, thanks to the DIGS, which can be considered as a major limitation given the central role of mood variability in AN psychopathology [64]. The role of depression on IGT performance is still debated, but the most recent systematic review on the role of depression on cognitive performance in AN mainly showed no influence of depressive symptoms on decision-making [54]. More recent studies replicate this finding [24, 65], and that differences in model fit between patients with AN and HC were not driven by baseline differences in depression [27]. We ruled out the influence of depression anyway, repeating all analyses after excluding patients with current depressive disorder according to the DIGS, and showing that results did not change.

A more consistent body of evidence supports a role for anxiety as a potential confounder of decision-making in eating disorders [65], with contrasting findings [27]. Unsurprisingly, A-AN and R-AN had the highest levels of anxiety. Even though separating anxiety from AN psychopathology remains delicate, given the high entanglement between the two syndromes [66], we excluded the contaminating effect of state anxiety on decision-making [67], by including this covariate in the ANCOVA.

Another limitation is represented by the disparity between the sample size of R-AN and that of the three other groups. This limitation may have affected significance of some results, but was sufficient to replicate results of previous literature, in particular, that of significant differences between acute and recovered patients. The relatively scarce number of R-AN patients is due to recruitment difficulties, but is in line from that of previous studies on the subject [15, 55]. Accordingly, the PVL model provided intriguing results that are complementary to, but do not explain, decision-making impairment in A-AN.

The cross-sectional design of the study limits the generalizability of results. It has been shown that A-AN is characterized by great interindividual variability in decision-making, with better performances predicting response to therapy [59]. A prospective evaluation is indispensable to disentangle the relationship between remission achievement and decision-making performance, and to confirm the impact of renutrition on the latter.

Finally, it could be criticized that no distinction has been made between restricting-type and binge-purging AN. On the other hand, previous studies found comparable performance between the two groups [18, 62] even though this result is still debated [7]. Nonetheless, altered performance at the IGT has been demonstrated in both subtypes [7] and additional analyses distinguishing the two groups were out of the scope of this research.

## Conclusion

Decision-making performance seems a state-associated cognitive feature of AN, prevalent at acute stages of the disease. Decreased sensitivity to feedback was found in both acute and remitted patients, suggesting trait-associated features. Decision-making process in AN may be the result of the interplay between trait-associated decisional features and state-associated compensatory mechanisms, allowing for unimpaired performance in some remitted patients.

Prospective studies are necessary to confirm that weight gain and reduction of symptoms facilitate a more appropriate decision-making process. These two dimensions could serve as important leverages for the improvement of cognitive functioning in patients with AN.

## Data Availability

The data that support the findings of this study are available from the corresponding author, upon reasonable request.

## Supplementary material

For supplementary material accompanying this paper visit https://doi.org/10.1192/j.eurpsy.2022.2327. click here to view supplementary material
