# Supplementary material for: Is decision-making impairment an endophenotype of anorexia nervosa?
Source: Eur Psychiatry. 2022 Oct 21;65(1):e68. doi: 10.1192/j.eurpsy.2022.2327 (PMC9677444; doi:10.1192/j.eurpsy.2022.2327)

Table S1: Performance at the Iowa Gambling Task (IGT) in a group of 114 patients with anorexia nervosa (91 acute, 23 remitted). Comparisons were made by ANCOVAs between patients taking psychotropic medication (benzodiazepines, antidepressants, antipsychotics) and patients who did not. National adult Reading Test (NART) score, Barratt impulsivity scale (BIS-10), age and STAI: State-Trait Anxiety Inventory (STAI)-A were introduced in the model as covariates.

|  | Yes | No | df | Mean sq sum | F | p-value |
| --- | --- | --- | --- | --- | --- | --- |
| **Benzodiazepines, n(%)** | 50 (43.85%) | 64 (56.15%) |  |  |  |  |
| IGT total raw score | 12.16 ± 30.08 | 13.42 ± 27.33 | 1 | 50.98 | .06 | .80 |
| PVL - A (learning) | .55 ± .24 | .51 ± .25 | 1 | .01 | .24 | .62 |
| PVL - alpha | .15 ± .04 | .16 ± .04 | 1 | <.01 | 2.01 | .16 |
| PVL - consistency | .67 ± .28 | .67 ± .33 | 1 | 3.23E-05 | <.01 | .98 |
| PVL - lambda | 1.61 ± 1.65 | 1.44 ± 1.71 | 1 | .46 | .16 | .69 |
| **Antidepressants, n(%)** | 39 (34.21%) | 75 (65.79%) |  |  |  |  |
| IGT total raw score | 11.08 ± 34.07 | 14.34 ± 25.36 | 1 | 265.90 | .31 | .58 |
| PVL - A (learning) | .54 ± .26 | .53 ± .23 | 1 | .03 | .56 | .46 |
| PVL - alpha | .15 ± .05 | .16 ± .03 | 1 | .01 | .63 | .43 |
| PVL - consistency | .70 ± .34 | .67 ± .28 | 1 | 7.444E-05 | <.01 | .98 |
| PVL - lambda | 1.34 ± 1.59 | 1.61 ± 1.71 | 1 | .76 | .27 | .61 |
| **Antipsychotics, n(%)** | 17 (14.91%) | 97 (85.09%) |  |  |  |  |
| IGT total raw score | 10.12 ± 31.89 | 12.86 ± 27.78 | 1 | 203.87 | .25 | .62 |
| PVL - A (learning) | .56 ± .20 | .53 ± .25 | 1 | .03 | .52 | .47 |
| PVL - alpha | .16 ± .03 | .16 ± .04 | 1 | 1.436E-05 | .01 | .92 |
| PVL - consistency | .63 ± .24 | .68 ± .31 | 1 | .19 | 2.34 | .13 |
| PVL - lambda | 1.94 ± 1.75 | 1.45 ± 1.66 | 1 | 4.16 | 1.46 | .23 |

IGT: Iowa gambling test IGT: Iowa gambling test; PVL: Prospect Valence Learning; df: degrees of freedom.

Figure S1: Mean Iowa Gambling Task (IGT) block scores of a group of patients with acute anorexia nervosa (A-AN), remitted anorexia nervosa (R-AN), healthy controls (HC) and unaffected relatives of patients with anorexia nervosa (UR). Means are adjusted for the covariates age, National Adult Reading Test (NART) score, Barratt Impulsivity Scale (BIS10) score and State-Trait Anxiety Inventory-A (STAI-A) score, measuring state anxiety.


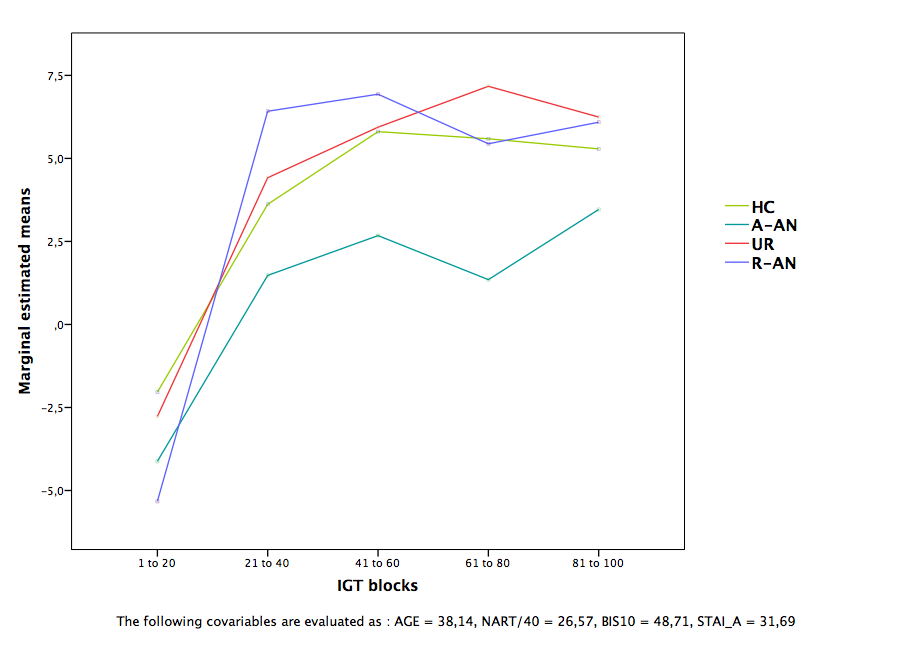

Supplement: Supplementary file 1 [file S0924933822023276sup001.docx]
